# Effect of Peptide Receptor Radionuclide Therapy on Somatostatin Receptor Status and Glucose Metabolism in Neuroendocrine Tumors: Intraindividual Comparison of Ga-68 DOTANOC PET/CT and F-18 FDG PET/CT

**DOI:** 10.1155/2011/524130

**Published:** 2011-11-09

**Authors:** Sowon Oh, Vikas Prasad, Dong Soo Lee, R. P. Baum

**Affiliations:** ^1^Department of Nuclear Medicine, College of Medicine, Seoul National University, Seoul 151-742, Republic of Korea; ^2^Department of Nuclear Medicine, Center for PET/CT, Zentralklinik Bad Berka, Robert Koch Allee 9, 99437 Bad Berka, Germany

## Abstract

The heterogeneous nature of the neuroendocrine tumors (NET) makes it challenging to find one uniformly applicable management protocol which is especially true for diagnosis. The discovery of the overexpression of somatostatin receptors (SMS-R) on neuroendocrine tumor cells lead to the generalized and rapid acceptance of radiolabeled somatostatin receptor analogs for staging and restaging of NET as well as for Peptide Receptor Radionuclide Therapy (PRRNT) using Y-90 and Lu-177 DOTATATE/DOTATOC. In this present work we tried to look in to the effect of PRRNT on the glucose metabolism assessed by F-18 FDG PET/CT and SMS-R density assessed by Ga-68 DOTANOC PET/CT. We observed a complex relationship between the somatostatin receptor expression and glucose metabolism with only 56% (77/138) of the lesions showing match, while the others show mismatch between the receptor status and metabolism. The match between receptor expression and glucose metabolism increases with the grade of NET. In grade 3 NET, there is a concurrence between the changes in glucose metabolism and somatostatin receptor expression. PRRNT was found to be more effective in lesions with higher receptor expression.

## 1. Introduction

Neuroendocrine tumours (NETs) are a rare but very heterogeneous group of neoplasms [1, 2]. NETs are generally slow-growing tumors having a relatively good prognosis. Although many of NETs are clinically silent, presenting with symptoms of mass effects or distant metastases [1–3], they often lead to debilitating symptoms like diarrhea and flush, which deteriorates patients' quality of life [4]. Surgery is the only treatment having curative intent, while cold somatostatin analogues are primarily used for reducing the symptoms [2, 4, 5]. However, the inhibition of secretion of the peptides responsible for symptoms is often transient as the median duration of improvement of carcinoid symptoms by octreotide is known to be over 12 months [6]. Furthermore, somatostatin analogues seldom have antitumor effect, specifically if more than 10% of the liver is involved with tumor [7–11]. 

Somatostatin receptor scintigraphy (SRS) is still considered as the gold standard for staging of NET. However, recent studies have clearly shown the superiority of Ga-68 somatostatin receptor PET/CT over SRS [12–14]. High tumor uptake on SRS is reported to be associated with better tumor response to PRRNT [15, 16] and that is the reason why tumor uptake on SRS has been suggested as a new prognostic factor in well-differentiated endocrine tumors [17, 18]. ^68^Ga DOTANOC has been shown to have a very good binding affinity to somatostatin receptor subtypes 2 and 3 and higher binding affinity to somatostatin receptor subtype 5 as compared to DOTATATE and DOTATOC [19]. On the other hand, ^18^F-fluorodeoxyglucose (FDG) has only limited value in good differentiated NET and is recommended only if SRS is negative especially in poorly differentiated NET [20, 21]. Additionally, ^68^Ga DOTATATE PET/CT has been shown to be a useful novel imaging modality for NETs and was found to be superior to ^18^F-FDG for imaging well-differentiated NET in previous study. However, functional imaging with both ^68^Ga DOTATATE and ^18^F-FDG has shown to address different biological properties of the neuroendocrine tumor lesions in patients planned for ^90^YDOTATOC PET/CT [22] and has been proposed as for comprehensive tumor assessment in intermediate- and high-grade tumors [23]. In our study, an intraindividual comparison was made between the somatostatin receptor status using ^68^Ga DOTANOC PET/CT and glucose metabolism using ^18^F FDG PET/CT after PRRNT.

## 2. Materials and Methods

### 2.1. Patient Population

Overall, 25 patients with progressive, metastasized neuroendocrine tumor were included. All the patients were imaged using FDG-PET/CT and ^68^Ga DOTANOC PET/CT.

The average time interval between the two different PET/CTs was 3 days (mean age of 58.7 ± 12.2 years; female to male ratio, 9 : 16). For precise lesion-by-lesion comparison and to avoid any partial volume effect, lesions larger than 2 cm in diameter were measured. The metastatic lesions that were too confluent or conglomerated to be separately analyzed were excluded from the study. Detailed demographic data are shown in Table 1.

### 2.2. Radiopharmaceutical Preparation

**^68^Ga DOTANOC Labeling**

To perform receptor PET/CT, ^68^Ga was eluted from ^68^Ge/^68^Ga generator (obtained from Eckert and Ziegler, Berlin, Germany), and the labeling process and associated quality control were performed according to the technique mentioned in a previous publication [24, 25].

**^90^Y DOTATATE and ^177^Lu DOTATATE**

Both ^90^Y DOTATATE and ^177^Lu DOTATATE were prepared in house, in a class 10 facility, under strict GMP protocol. The radiochemical purity (checked using HPLC) of the final preparation was always >99%. The total amount of peptide administered in each cycle of ^90^Y DOTATATE and ^177^Lu DOTATATE ranged from 75 to 250 *μ*g and from 160 to 500 *μ*g respectively.

### 2.3. Imaging Protocols

A dual-modality PET/CT tomography (Biograph duo; Siemens Medical Solutions) was used, which consists of a PET system with a full-ring lutetium oxyorthosilicate (LSO) and a CT component corresponding to a Somatom Emotion Duo (Siemens Medical Solutions), a 2-row spiral CT system with a maximum continuous scan time of 100 s and a maximum rotation speed of 75 rpm. Water-equivalent iodine containing contrast agent was given intravenously at 60 minutes before starting the ^68^Ga DOTANOC PET/CT. This dispersion is routinely used in PET/CT without known adverse side effects on the accumulation of ^68^Ga DOTANOC. For ^18^F-FDG scan, fasting for more than 12 hours was additionally requested. In addition, urine voiding was asked to all patients immediately before the PET/CT. The image acquisition started 60 minutes after administration of approximately 120 MBq of ^68^Ga DOTANOC and 250 MBq ^18^F-FDG, while the patients were positioned head first supine with the arms raised in accordance with a standard CT practice and following the EANM guidelines [26, 27]. The acquisition parameters were kept strictly the same for pre- and post-PRRNT PET/CT imaging.

A topogram was acquired over 1.024 mm axially, and coaxial whole-body imaging ranges were defined on the topogram from the skull to the upper thighs (7-8 PET bed positions, or 90–100 cm, depending on the size of the patient). Intravenous radioiodine contrast media of 100 milileters was injected by an automated injection pump after checking the topogam, and contrast-enhanced CT images were acquired during venous phase of the contrast from the level of the skull to the thighs with a 30 s delay CT in spiral mode using a continuous acquisition at 130 kVp, 115 mAs, 4 mm collimation, 5 mm slice width, a table feed of 8 mm per rotation at 0.8 s rotation time, and 2.4 mm slice spacing. For the CT image acquisition, a limited breath hold protocol was required to the patients. Then PET emission scan was obtained in 3-dimensional mode at each bed position for 1-2 minutes (depending upon the weight and height of the patient) for the same coverage with the CT images, which started in the caudocranial direction after the patients moving automatically to the PET toward the rear of the gantry. As a result, a total emission scan time did not exceed 24 minutes, and a total PET/CT examination took about 30 minutes including patient positioning, CT, and PET imaging.

### 2.4. Therapy Protocol

The legal requirements were met in accomplishing the study (including ethical and local radiation protection regulations). The study was performed according to guidelines, approved by the local ethical committee at the Zentralklinik Bad Berka and in accordance with German regulations (as published by the Federal Office for Radiation Protection, BfS) concerning radiation safety. Written informed consent was obtained from all patients before therapy. All patients, who were enrolled in this study, presented with progressive neuroendocrine tumors after having exhausted all conventional therapeutic options. Among other factors (Table 1), the degree of somatostatin receptor expression (maximum Standardized Uptake Value SUV_max_) as determined by Ga-68 somatostatin receptor PET/CT [28, 29], number and size of metastases, pretherapy renal function, and hematological profile was primarily taken into consideration before estimation of the dose to be administered, thereby individualizing the patients' therapy. 

On an average, each patient received 3.0 GBq ^90^Y DOTATATE and/or 7.5 GBq ^177^Lu DOTATATE per cycle. In total, 17 patients were treated with only one cycle of PRRNT, while the remaining 8 patients received upto three cycles.

### 2.5. Renal Protection

For kidney protection, every patient was coinfused with 2000 mL of a renoprotective amino acid mixture of 5% Lysine HCL and 10% L-Arginine HCL in 250 mL NaCl at pH 7.4 and osmolarity of 400 mosmol/L. This infusion was started 30 minutes prior to the administration of the therapeutic dose and continued for 4 hours. The radiopharmaceutical was coadministered over 10–15 minutes by using a second infusion pump system [30]. Each patient was well hydrated by ensuring an intake of at least 1 litre of mineral water before therapy and 2-3 litres thereafter. In case there was evidence of renal obstruction (physiologic or pathological), i.v. injection of 20–40 mg of furosemide in 1.5-2 litre of deltajonin was given over 2–4 hours after therapy.

### 2.6. Data Reconstruction

For attenuation correction of the PET emission images, the CT transmission data were used. The PET images were reconstructed iteratively using an attenuation-weighted ordered-subsets expectation maximization algorithm with 2 iterations and 8 subsets on 128 × 128 matrices and with a 5-minute Gaussian postreconstruction filtering.

### 2.7. Image Evaluation and Analysis

For the analysis of PET/CT, two different imaging viewing systems were used by an experienced radiologist and two experienced nuclear medicine physicians, respectively; a Syngo viewer for the image analysis of CT and an E. soft workstation (Siemens Medical Solutions) for the PET/CT image assessment. At first, the maximum intensity projection (MIP) images were visually examined in varying scales, and then each single transverse slice was looked over from the skull base to the midthighs in combination with the corresponding CT image and the fused image slice. Each lesion showing a focal abnormal tracer uptake was recorded by a slice number and anatomic localization, and any lesion with intensity greater than background which could not be explained by physiological activity was considered to be indicative of tumor tissue. For semiquantitative analysis of the lesions, a region of interest (ROI) automatically drown around each lesion maximum standardized uptake value SUV_max_ was evaluated. Up to a maximum of five lesions per organ and 10 lesions in total were chosen in SUV_max_ order to avoid bias of statistical counting. Changes in the size on CT (ΔCT), SUV_max_ of FDG (ΔFDG), and SUV_max_ of ^68^Ga DOTANOC (ΔSMS-R) were compared with each other.

Additionally, all the lesions were regrouped based upon the tumor location-primary sites, liver, lymph nodes, and bones.

### 2.8. Statistical Analysis

SUV_max_ of each lesion prior to and after PRRNT were compared with Student's *t*-test. In case that the total number of cases was nonparametric, Wilcoxon signed-rank test and Mann-Whitney *U* test were adopted. Analysis of variance test was done to compare means of two groups. A value of *P *< 0.05 was considered significant. All statistical analysis was carried out using MedCalc statistical software (version 9.3.2; MedCalc, Mariakerke, Belgium).

## 3. Results

### 3.1. Analysis Based on the Uptake Pattern of Two PET/CTs

**Figure 1:**
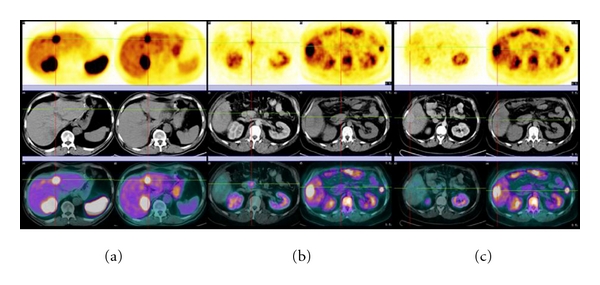
Grouping based on the uptake of two PET/CTs. (a) Group A: a metastatic liver lesion presented both SMS (on the left) and FDG uptake (on the right). (b) Group B: a primary tumor in the pancreas head presented SMS uptake (on the left), FDG PET/CT images on the right showed no uptake in the primary. (c) Group C: a metastatic liver lesion presented FDG uptake (on the right) but was negative for somatostatin receptor expression (on the left).

In 25 patients, a total of 133 discrete lesions were identified on either ^68^Ga DOTANOC (SMS) PET/CT or ^18^F FDG (FDG) PET/CT. Most lesions (*n* = 126, 94.7%) were positive on SMS PET/CT, but 61.9% (*n* = 78) was positive on FDG PET/CT at the same time. The remaining lesions (*n* = 7, 5.3%) were only detectable by FDG PET/CT and were negative on SMS PET/CT. The lesions were subclassified as follows: both SMS as well as FDG positive (group A: 58.6%, *n* = 78), only SMS positive (group B: *n* = 38, 28.6%), or FDG positive (group C: *n* = 7, 5.3%). The PET/CT findings of the three groups are shown in Figure 1.

In patients where Ki-67 was available (*n* = 10), 6 had Ki-67 >20% (grade 3 NET G3), 2 had Ki-67 between 3 and 20 (grade 2 NET G2), and 2 had Ki-67 <2% (grade 1 NET, G1). In this subgroup of patients, FDG could pick up 67% of the receptor-positive lesions (40/61); in G3 NET, FDG PET could pick up 90% of the lesions (36/39). There was a significant correlation between the ΔFDG and ΔSMS-R. There was significant correlation between (32 measurable lesions on CT also positive on FDG PET and SMS-R PET) the ΔFDG and ΔCT (*P* = 0.027), between ΔFDG and ΔSMS-R (0.007), but no significant change between the ΔSMS-R and ΔCT. 

**Figure 2:**
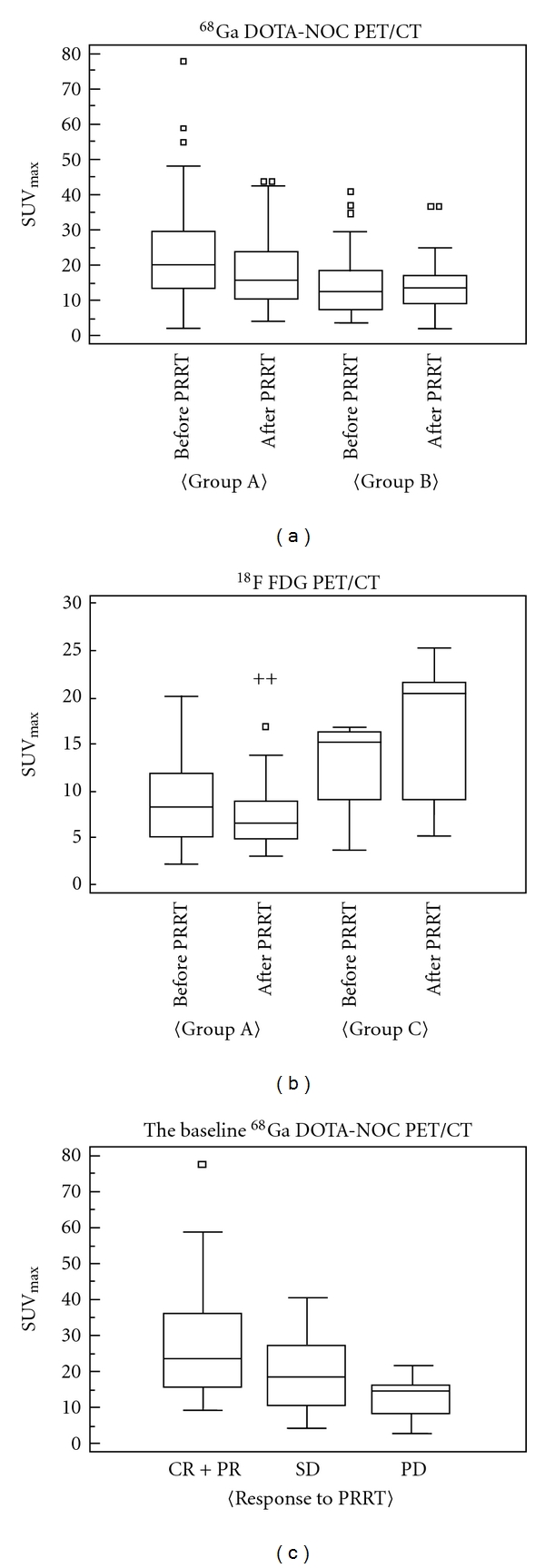
Relationship between SUV_max_ before and after PRRNT. (a) Changes of SUV on ^68^Ga DOTANOC (SMS) PET/CT in response to PRRNT. The SMS uptake decreased significantly in both group A and group B; baseline SUV_max_ of group A was higher than that of group B. (b) SUV changes on ^18^F FDG (FDG) PET/CT in response to PRRNT. The FDG uptake decreased significantly in group A, whereas the uptake in group C increased. The baseline SUV_max_ of group C tended to be higher than that of group A. (c) The relationship between therapy response and baseline SUV_max_ of ^68^Ga DOTANOC PET/CT. The response is positively correlated with the baseline SUV_max_.

In group A, the SUV_max_ of SMS (23.0 ± 13.6 versus 17.3 ± 9.4, *P* < 0.0001) (see Table 2 and Figure 2) as well as the SUV_max_ of FDG (9.0 ± 4.3 versus 6.6 ± 4.2, *P* < 0.0001) decreased significantly after PRRNT. The SUV_max_ of SMS also decreased in group B (14.7 ± 9.1 versus 11.5 ± 8.8, *P *< 0.0001), but the SUV_max_ of FDG in group C (12.6 ± 5.0 versus 16.9 ± 8.0, *P* = 0.0469) increased after PRRNT. As for the radiotracer uptake pattern, the baseline SUV_max_ of SMS in group A was significantly higher than that of group B (23.0 ± 13.6 versus 14.7 ± 9.1,* P *= 0.0003). On the other hand, the baseline SUV_max_ of FDG in group A tended to be lower than in group C (9.0 ± 4.3 versus 12.6 ± 5.0) though it failed to reach a statistical significance.

### 3.2. Analysis Based on the Tumor Location

The lesions were regrouped based on the location of the tumor-primary site (*n* = 19, 14.3%), lymph nodes metastases (*n* = 26, 19.5%), liver metastases (*n* = 71, 53.4%), and bone metastases (*n* = 17, 12.8%). The lesions were further classified according to the receptor expression and FDG uptake; matched group contained lesions showing somatostatin receptor expression and FDG uptake, and the mismatched group contained lesions which were either somatostatin receptor-positive lesion or FDG positive. In the liver, FDG PET/CT found additional lesions (*n* = 7) which did not accumulate Ga-68 DOTANOC. All the other lesions were somatostatin receptor positive.

The pattern of SMS and FDG changes of the tumor in response to PRRNT differed by its location (see Table 3). The receptor expression as detected by SUV_max_ in the primary tumor (23.2 ± 11.0 versus 17.6 ± 8.0,* P* = 0.037) and lymph nodes (21.3 ± 13.6 versus 15.3 ± 9.6,* P *< 0.0001) decreased significantly after PRRNT, whereas the corresponding FDG did not show any significant change. In the mismatched groups of the primary tumor and lymph nodes, no significant change was seen in the SMS uptake after the PRRNT application; the baseline SUV_max_ were lower than those of matched groups (primary tumor: 14.5 ± 13.5 versus 23.2 ± 11.0,* P *= 0.043, lymph nodes: 9.5 ± 5.1 versus 21.3 ± 13.6,* P *= 0.018). On the contrary, metastatic bone lesions showed a significant decrease of the Ga-68 DOTANOC uptake only in the mismatched group (7.5 ± 3.1 versus 5.1 ± 3.1,* P *= 0.007). The FDG uptake of the bone lesions also decreased significantly (8.6 ± 3.2 versus 3.2 ± 1.6,* P *= 0.007). In the matched group, the bone lesions which were also FDG avid did not show any significant decrease on receptor expression on SMS-R PET. In the liver, the changes of each PET/CT were different among the three subgroups. The matched group of the liver showed significant decreases in both SMS uptake (26.5 ± 13.9 versus 19.6 ± 9.5,* P *= 0.0001) and FDG uptake (8.8 ± 4.2 versus 6.2 ± 3.6,* P *< 0.0001). In the mismatched group, the SUV_max_ of the liver lesions showing receptor expression decreased after PRRNT (19.0 ± 7.4 versus 14.9 ± 5.7,* P *= 0.0023), whereas the SUV_max_ of the hepatic lesions showing only FDG uptake increased (12.6 ± 5.0 versus 16.9 ± 8.0,* P *= 0.0469).

## 4. Discussion

It is known that NETs except benign insulinomas highly express SMS with an incidence of 80–100%, and its density is also predominantly high [31]. In this study, most lesions were well visualized on SMS PET/CT, and more than half of the lesions showed response by means of PRRNT. Also, higher tumor remission rate was correlated with a high-baseline SUV_max_ on SMS PET/CT. This finding is well consistent with previous studies [16, 32], and PRRNT seems to be quite an effective therapy option for NET patients expressing adequate densities of SMS on the tumors. 

To the best of our knowledge, this study has shown for the first time that PRRNT seemed to have an effect on glucose metabolism of NETs showing somatostatin receptor expression. It is well known that the changes in the FDG correlate significantly with tumor response [33]. Because the ΔFDG showed significant correlation with the ΔSMS, the changes in ΔSMS could be taken as a measure of the response assessment. 

This study also showed, as expected, that PRRNT is only effective in lesions showing adequate SMS expression. This was the reason why receptor-negative hypermetabolic lesions progressed after PRRNT. 

The main use of FDG PET in diagnosis of NETs depends on the grade of differentiation and/or aggressiveness of NETs. In our patient population with proliferation rate of more than 20%, a very high percentage of receptor-positive lesions were also picked up by FDG PET. Interestingly, nearly all the measured lesions showing FDG uptake, even in patients with high proliferation rate, showed good response to PRRNT on CT as well as on SMS-R PET. Similar results have been observed by Ezziddin et al. where the authors showed that the response to PRRNT is very good even if the proliferation rate is roughly 20% [34]. This observation supports the use of PRRNT in somatostatin receptor-positive NET. However, there is as of yet no consensus or literature comparing PRRNT with chemotherapy in grade 3 NET.

 One of the most interesting observations in this study was the difference in the pattern of response to PRRNT based on the tumor localization. The response to PRRNT was indifferent to the glucose metabolism in the lymph node metastases, liver metastases, and the primary tumor. However, the hypermetabolic bone lesions did not show any significant response to PRRNT. This difference could be related to the different biological properties of tumor cells in bone or bone marrow as compared to those metastasizing to liver or lymph nodes. This observation needs to be validated in further studies.

## 5. Conclusion

There is a complex relationship between the somatostatin receptor expression and glucose metabolism with only 56% (77/138) of the lesions showing match, while the others show mismatch between the receptor status and metabolism. The match between receptor expression and glucose metabolism increases with the grade of NET. In grade 3 NET, there is a concurrence between the changes in glucose metabolism and somatostatin receptor expression. PRRNT is more effective in lesions with higher receptor expression.

## Figures and Tables

**Table 1:** Demographics and clinical characteristics.

Characteristics	No.
Age	
Mean ± SD	58.7 ± 12.2
Sex, *n* (%)	
Male	16 (64%)
Female	9 (36%)
Pathology	
GEP-NET	18
CUP	2
Carcinoid	4
Pheochromocytoma	1
Number of PRRT	
1	17
up to 3	8

GEP-NET: gastroenteropancreatic neuroendocrine tumor.

**Table 2:** Response to PRRNT: comparison of SMS-R and FDG PET/CT.

Group	PET/CT	Response	Before PRRT		After PRRT	
			*N*	SUV_max_	*N *	SUV_max_^†^
Group A	SMS	CR + PR	44	27.4 ± 14.9	41	16.3 ± 9.9^†^
		SD	24	19.1 ± 9.9	24	18.0 ± 9.5^†^
		PD	10	12.9 ± 5.6	10	19.9 ± 7.5*
		Total	78	23.0 ± 13.6	75	17.3 ± 9.4^†^
	FDG	CR + PR	44	9.4 ± 4.4	37	6.5 ± 4.1^†^
		SD	24	8.2 ± 3.7	20	6.3 ± 3.2*
		PD	10	8.9 ± 5.1	7	7.1 ± 6.6
		Total	78	9.0 ± 4.3	64	6.6 ± 4.2

Group B	SMS	CR + PR	25	12.9 ± 9.3	15	6.7 ± 6.5^†^
		SD	20	17.4 ± 8.9	20	16.5 ± 8.4*
		PD	3	12.0 ± 5.2	3	18.4 ± 5.8
		Total	48	14.7 ± 9.1	38	11.5 ± 8.8

Group C	FDG	Total	7	12.6 ± 5.0	7	16.9 ± 8.0*

^†^: *P* < 0.0001, *: *P* < 0.05.

**Table 3:** Different response patterns according to tumor location.

Location	Group	PET/CT	Before PRRT		After PRRT	
			*N*	SUV_max_	*N*	SUV_max_
Primary sites	Mat	SMS	10	23.2 ± 11.0	9	17.6 ± 8.0*
		FDG	10	9.0 ± 4.2	9	8.2 ± 4.2
	Mis	SMS	9	14.5 ± 13.5	5	12.9 ± 14.7

LN	Mat	SMS	17	21.3 ± 13.6	17	15.3 ± 9.6^†^
		FDG	17	9.5 ± 5.1	16	8.4 ± 5.3
	Mis	SMS	9	10.5 ± 5.1	5	7.5± 7.4

Liver	Mat	SMS	42	26.5 ± 13.9	39	19.6 ± 9.5^†^
		FDG	42	8.8 ± 4.2	32	6.2 ± 3.6^†^
	Mis	SMS	22	19.0 ± 7.4	21	14.9 ± 5.7*
		FDG	7	12.6 ± 5.0	7	16.9 ± 8.0*

Bone	Mat	SMS	9	9.9 ± 3.5	9	9.6 ± 4.9
		FDG	9	8.6 ± 3.2	7	3.2 ± 1.6*
	Mis	SMS	8	7.5 ± 3.1	7	5.1 ± 3.1*

Abbreviations: Mat: matched, Mis: mismatched, LN: lymph nodes.

^†^: *P* < 0.0001, *: *P* < 0.05.

## References

[1] Barakat MT, Meeran K, Bloom SR (2004). Neuroendocrine tumours. *Endocrine-Related Cancer*.

[2] Modlin IM, Mark K, Latich I, Zikusoka MN, Shapiro MD (2005). Current status of gastrointestinal carcinoids. *Gastroenterology*.

[3] Kaltsas GA, Besser G, Grossman AB (2004). The diagnosis and medical management of advanced neuroendocrine tumors. *Endocrine Reviews*.

[4] Oberg K, Eriksson B (1989). Medical treatment of neuroendocrine gut and pancretic tumors. *Acta Oncologica*.

[5] Kwekkeboom DJ, Krenning EP, Lebtahi R (2009). ENETS consensus guidelines for the standards of care in neuroendocrine tumors: peptide receptor radionuclide therapy with radiolabeled somatostatin analogs. *Neuroendocrinology*.

[6] Kvols LK, Moertel C, O’Connell MJ (1986). Treatment of the malignant carcinoid syndrome. Evaluation of a long-acting somatostatin analogue. *The New England Journal of Medicine*.

[7] Rinke A, Muller HH, Schade-Brittinger C (2009). Placebo-controlled, double-blind, prospective, randomized study on the effect of octreotide LAR in the control of tumor growth in patients with metastatic neuroendocrine midgut tumors: a report from the PROMID study group. *Journal of Clinical Oncology*.

[8] Faiss S, Pape U, Bohmig M (2003). Prospective, randomized, multicenter trial on the antiproliferative effect of lanreotide, inteferon alfa, and their combination for therapy of metastatic neuroendocrine gastroenteropancreatic tumors—the international lanreotide and interferon alfa study group. *Journal of Clinical Oncology*.

[9] Janson ET, Oberg K (1993). Long-term management of the carcinoid syndrome. Treatment with octreotide alone and in combination with *α*-interferon. *Acta Oncologica*.

[10] Panzuto F, Di Fonzo M, Iannicelli E (2006). Long-term clinical outcome of somatostatin analogues for treatment of progressive, metastatic, well-differentiated entero-pancreatic endocrine carcinoma. *Annals of Oncology*.

[11] Shojamanesh H, Gibril F, Adeline L (2002). Prospective study of the antitumor efficacy of long-term octreotide treatment in patients with progressive metastatic gastrinoma. *Cancer*.

[12] Buchmann I, Henze M, Engelbrecht S (2007). Comparison of 68Ga-DOTATOC PET and 111In-DTPAOC (Octreoscan) SPECT in patients with neuroendocrine tumours. *European Journal of Nuclear Medicine and Molecular Imaging*.

[13] Hofmann M, Maecke H, Borner A (2001). Biokinetics and imaging with the somatostatin receptor PET radioligand 68Ga-DOTATOC: preliminary data. *European Journal of Nuclear Medicine*.

[14] Kowalski J, Henze M, Schuhmacher J, Helmut RM, Michael H, Uwe H (2003). Evaluation of positiron emission tomography imaging using [^68^Ga]-DOTA-D Phe(1)-Tyr(3)-Octreotide in comparison to [^111^In]-DTPAOC SPECT. First results in patients with neuroendocrine tumors. *Molecular Imaging & Biology*.

[15] Kwekkeboom DJ, Bakker W, Kam BL (2003). Treatment of patients with gastro-entero-pancreatic (GEP) tumours with the novel radiolabelled somatostatin analogue [^177^Lu-DOTA^0^,Tyr^3^]octreotate. *European Journal of Nuclear Medicine and Molecular Imaging*.

[16] Kwekkeboom DJ, de Herder WW, Kam BL (2008). Treatment with the radiolabled somatostatin analog [^177^Lu-DOTA^o^,Tyr^3^] octreotate: toxicity, efficacy and survival. *Journal of Clinical Oncology*.

[17] Haug AR, Auernhammer CJ, Schmidt GP (2010). 68Ga-DOTATATE PET/CT for the early prediction of response to somatostatin receptor-mediated radionuclide therapy in patients with well-differentiated neuroendocrine tumors. *Journal of Nuclear Medicine*.

[18] Asnacios A, Courbon F, Rochaix P (2008). Indium-111-pentetreotide scintigraphy and somatostatin receptor subtype 2 expression: new prognostic factors for malignant well-differentiated endocrine tumors. *Journal of Clinical Oncology*.

[19] Wild D, Schmitt JS, Ginj M (2003). DOTA-NOC, a high-affinity ligand of somatostatin receptor subtypes 2, 3 and 5 for labelling with various radiometals. *European Journal of Nuclear Medicine and Molecular Imaging*.

[20] Adams S, Baum R, Rink T, Usadel KH (1998). Limited value of fluorine-18 fluorodeoxyglucose positron emission tomography for the imaging of neuroendocrine tumours. *European Journal of Nuclear Medicine*.

[21] Pasquali C, Rubello D, Sperti C (1998). Neuroendocrine tumor imaging: can ^18^F-fluorodeoxyglucose positron emission tomography detect tumors with poor prognosis and aggressive behavior?. *World Journal of Surgery*.

[22] Koukouraki S, Strauss LG, Georgoulias V, Eisenhut M, Haberkorn U, Dimitrakopoulou-Strauss A (2006). Comparison of the pharmacokinetics of 68Ga-DOTATOC and [ 18F]FDG in patients with metastatic neuroendocrine tumours scheduled for 90Y-DOTATOC therapy. *European Journal of Nuclear Medicine and Molecular Imaging*.

[23] Kayani I, Bomanji JB, Groves A (2008). Functional imaging of neuroendocrine tumors with combined PET/CT using 68Ga-DOTATATE (Dota-DPhe1, Tyr3-octreotate) and 18F-FDG. *Cancer*.

[24] Zhernosekov KP, Filosofov DV, Baum RP (2007). Processing of generator-produced ^68^Ga for medical application. *Journal of Nuclear Medicine*.

[25] Meyer GJ, Schuhmacher J, Knapp WH, Hofmann M (2004). ^68^Ga-labelled DOTA-derivatised peptide ligands. *European Journal of Nuclear Medicine and Molecular Imaging*.

[26] Virgolini I, Ambrosini V, Bomanji JB (2010). Procedure guidelines for PET/CT tumour imaging with 68Ga-DOTA-conjugated peptides: 68Ga-DOTA-TOC, 68Ga-DOTA-NOC, 68Ga-DOTA-TATE. *European Journal of Nuclear Medicine and Molecular Imaging*.

[27] Boellaard R, O’Doherty MJ, Weber WA (2010). FDG PET and PET/CT: EANM procedure guidelines for tumour PET imaging: version 1.0. *European Journal of Nuclear Medicine and Molecular Imaging*.

[28] Baum RP, Prasad V, Hommann M, Horsch D (2008). Receptor PET/CT imaging of neuroendocrine tumors. *Recent Results in Cancer Research. Fortschritte der Krebsforschung*.

[29] Prasad V, Ambrosini V, Hommann M, Hoersch D, Fanti S, Baum RP (2010). Detection of unknown primary neuroendocrine tumours (CUP-NET) using 68Ga-DOTA-NOC receptor PET/CT. *European Journal of Nuclear Medicine and Molecular Imaging*.

[30] Rimpler A, Barth I, Baum RB, Senftleben S, Geworski L (2008). *β* radiation exposure of staff during and after therapies with 90Y-labelled substances. *Radiation Protection Dosimetry*.

[31] Reubi JC, Kvols LK, Waser B (1990). Detection of somatostatin receptors in surgical and percutaneous needle biopsy samples of carcinoids and islet cell carcinomas. *Cancer Research*.

[32] Kwekkeboom DJ, Teunissen JJ, Bakker WH (2005). Radiolobeled somatostatin analog [^177^Lu-DOTA^0^,Tyr^3^]octreotate in patients with endocrine gastroenteropancreatic tumors. *Journal of Clinical Oncology*.

[33] Baum RP, Prasad V, Cook GJR, Maisey MN, Britton KE, Chengazi V (2006). Monitoring treatment. *Clinical Nuclear Medicine*.

[34] Ezziddin S, Opitz M, Attassi M (2010). Impact of the Ki-67 proliferation index on response to peptide receptor radionuclide therapy. *European Journal of Nuclear Medicine and Molecular Imaging*.

